# IRF5 promotes the proliferation of human thyroid cancer cells

**DOI:** 10.1186/1476-4598-11-21

**Published:** 2012-04-16

**Authors:** Michele Massimino, Paolo Vigneri, Manuela Fallica, Annamaria Fidilio, Alessandra Aloisi, Francesco Frasca, Livia Manzella

**Affiliations:** 1 Department of Clinical and Molecular Bio-Medicine, University of Catania, Via Androne, 83-95124, Catania, ITALY

**Keywords:** IRF5, Thyroid Cancer, Cell Proliferation

## Abstract

**Background:**

Interferon Regulatory Factor 5 is a transcription factor that regulates the expression of genes involved in the response to viral infection and in the stimulation of the immune system. Moreover, multiple studies have demonstrated that it negatively regulates cell growth and oncogenesis, favoring cell differentiation and apoptosis.

Thyroid carcinoma represents 98% of all thyroid malignancies and has shown a steady increase in incidence in both the USA and western European countries.

**Findings:**

We investigated the expression, localization and function of IRF5 in thyroid cancer cells and found that it is highly expressed in both primary and immortalized thyroid carcinomas but not in normal thyrocytes. IRF5 levels were variably modulated by Interferon alpha but IRF5 only localized in the cytoplasmic compartment, thus failing to induce p21 expression as previously reported in different cell models. Furthermore, ectopic IRF5 increased both the proliferation rate and the clonogenic potential of malignant thyroid cells, protecting them from the cytotoxic effects of DNA-damaging agents. These results were directly attributable to IRF5, as demonstrated by the reduction in colony-forming ability of thyroid cancer cells after IRF5 silencing. An IRF5-dependent induction of endogenous B-Raf observed in all thyroid cancer cells might contribute to these unexpected effects.

**Conclusions:**

These findings suggest that, in thyroid malignancies, IRF5 displays tumor-promoting rather than tumor-suppressor activities.

## Background

Interferon Regulatory Factor 5 plays essential roles in the regulation of genes induced by viral infection, cell growth, oncogenesis and apoptosis [1-8]. IRF5 was identified as a regulator of type I Interferon [9] and further studies revealed that IRF5 displays some tumor-suppressor properties as it can induce *p21, Bak, Bax*, and *Caspase 8*[10-12].

Thyroid carcinoma represents a unique model to study human carcinogenesis because it comprises tumors with different clinical and histological features [13]. Indeed, papillary and follicular thyroid cancers are slow-growing, well differentiated tumors, whereas anaplastic thyroid cancers are undifferentiated neoplasias that behave much more aggressively, usually leading to the death of the patient within one year from diagnosis [14,15].

We have analyzed the expression and function of IRF5 in thyroid carcinoma cells and report here multiple evidence suggesting that IRF5 may contribute to thyroid cancer proliferation and survival.

## Findings

### IRF5 is expressed in human thyroid cancer cells and is variably modulated by IFNα

**Figure 1:**
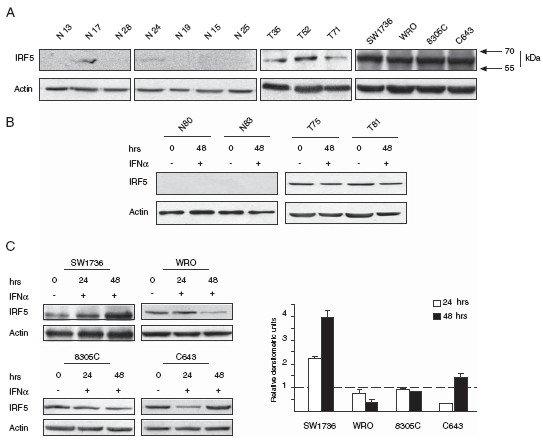
**IRF5 is expressed in neoplastic thyrocytes. A.** Specimens obtained from seven normal (N) thyrocytes, three patients with thyroid cancer (T) and the indicated immortalized cell lines were analyzed for IRF5 expression. Lysates from each sample were subjected to immunoblot using an anti-IRF5 antibody (Abcam). **B.** Two normal (N) and two neoplastic thyroid specimens (T) were treated with IFNα and subsequently analyzed by SDS-PAGE with the same antibody employed in A. **C.** Lysates from the specified cell lines were used to perform anti-IRF5 immunoblots (left panels) before and after exposure to IFNα (Sigma) for the indicated times. Densitometric analysis of the corresponding immunoblots is depicted in histograms (right panel) representing IRF5 expression after 24 (white columns) or 48 hour (black columns) of IFNα treatment. IRF5 levels in untreated cells are arbitrarily set at 1 (dashed line). Columns represent average ± standard deviation of three independent experiments.

To investigate IRF5 expression in both normal and neoplastic thyrocytes, we collected specimens derived from seven normal thyroids and three thyroid carcinomas as previously described [16]. We also examined four immortalized thyroid cancer cell lines (SW1736, WRO, 8305C and C643) all cultured as reported elsewhere [16]. An anti-IRF5 immunoblot found low or undetectable expression in normal thyrocytes. On the contrary, primary and immortalized thyroid cancer cells expressed high levels of IRF5, suggesting a possible role for this protein in thyroid carcinogenesis (Figure 1A). Interferon alpha (IFNα) is a well-known transcriptional inducer of IRF5 [17]. However, its efficacy on IRF5 induction in thyroid cancer cells is still unknown. We therefore exposed normal and neoplastic thyrocytes or the above-indicated thyroid cell lines to 1000U/mL IFNα and performed anti-IRF5 immunoblots. Unexpectedly, IFNα failed to induce IRF5 in primary thyrocytes (Figure 1B) causing instead modest variations in 8305C cells and stronger reductions - albeit with different kinetics - in WRO and C643 (Figure 1C). SW1736 were the only thyroid cancer line that displayed a robust up-regulation in IRF5 expression after IFNα.

### IRF5 lacks tumor-suppressor activity in thyroid cancer cells

**Figure 2:**
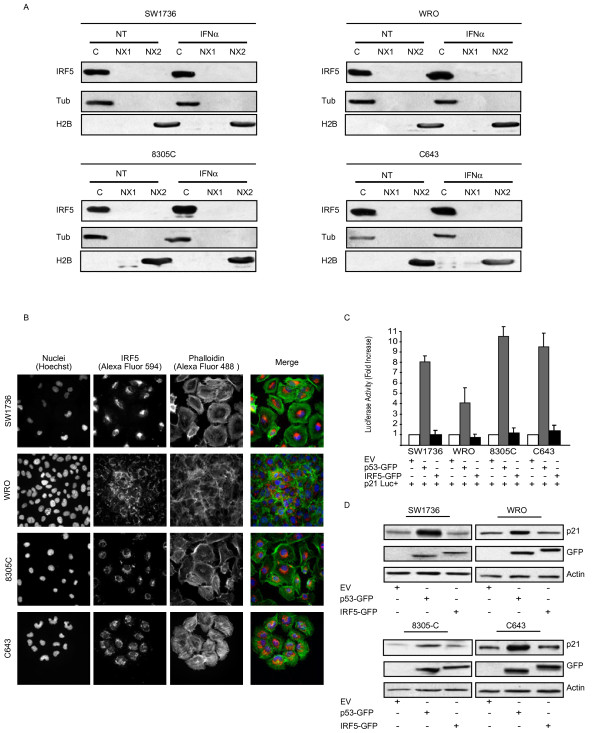
**IRF5 localizes in the cytoplasm and lacks tumor-suppressor activity in thyroid cancer cells. A.** Cytoplasmic (C), soluble (NX1) and insoluble (NX2) nuclear fractions were isolated from the indicated cell lines before and after a 24 hour treatment with IFNα. The different lysates were then blotted for IRF5. Tubulin (Tub) and histone 2B (H2B) (both from Santa Cruz) confirmed the purity of cytoplasmic and nuclear extracts. **B.** The same cells were subjected to IF for IRF-5 using the indicated secondary antibody. Hoechst and phalloidin (Alexa Fluor 488) were employed to identify the nuclear and cytoplasmic compartments, respectively. **C.** Thyroid cancer cell lines were also co-transfected with a reporter construct for p21 and p53-GFP or IRF5-GFP. After 48 hours, lysates were assayed for their relative luciferase activity expressed as fold activation over control cells (p21Luc + EV, arbitrarily set at 1). Results shown represent the average of three independent experiments. **D.** Alternatively, the same co-transfected cells were blotted using either anti-p21 (Santa Cruz) or anti-GFP antibodies (Covance), the latter to visualize either p53 or IRF5.

At steady state, IRF5 localizes to the cell cytoplasm. However, two Nuclear Localization Signals and a Nuclear Export Signal regulate IRF5 subcellular localization [18], promoting its nuclear import after viral infection [19]. To assess IRF5 intracellular distribution, we isolated nuclear and cytoplasmic fractions from four thyroid cancer cells using the Qproteom Nuclear Protein Kit (Qiagen). Immunoblots showed that IRF5 localized in the cytoplasmic compartment of each cell line, regardless of IFNα treatment (Figure 2A). These findings were further confirmed by immunofluorescence (IF) experiments carried out before and after IFNα treatment (Figure 2B and Additional file 1).

We next wanted to establish the transcriptional activity of IRF5 on genes involved in cell-cycle progression. SW1736, WRO, 8305C, and C643 were co-transfected (using Fugene-6 from Roche) with p21-Luc (p21 promoter driving the luciferase reporter gene) and GFP-IRF5 variant 3 (kindly provided by P.M. Pitha, Baltimore, MD), p53-GFP (positive control), or an empty vector (EV, negative control). Luciferase activity was then analyzed with the Dual-Luciferase® Reporter Assay kit (Promega). Unlike p53, IRF5 failed to induce p21-Luc activity (Figure 2C) as confirmed by anti-p21 immunoblots (Figure 2D).

### IRF5 induces the proliferation of thyroid cancer cells and rescues them from the effects of cytotoxic compounds

**Figure 3:**
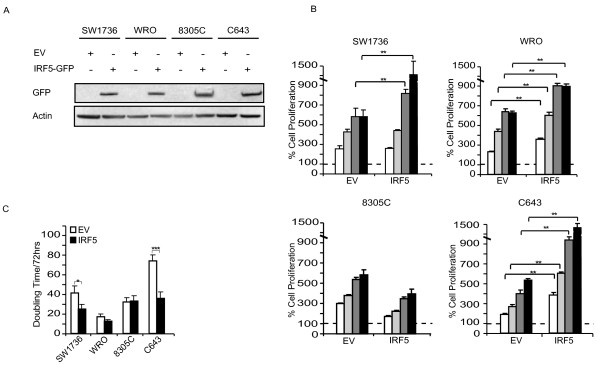
**IRF5 promotes thyroid cancer cells proliferation. A.** The specified cell lines were transduced with either EV or IRF5-GFP and analyzed for infection efficiency with an anti-GFP immunoblot. **B.** 5 × 10^3^ thyroid cancer cells described in A were plated in the absence of drugs for 24 (white columns), 48 (light grey columns), 72 (dark grey columns) and 96 hours (black columns) and analyzed for their proliferation. Dashed lines indicate baseline proliferation rates for each cell line assessed 4 hours after plating and arbitrarily set at 100%. **C.** The same thyroid cancer cells were implanted in 24-well plates and, after 72 hours, employed to calculate their doubling time using the doubling time online calculator (http://www.doubling-time.com/compute.php). *p-values* with 95% confidence intervals were obtained using *t*-tests determined with the Prism Software (GraphPad Software, *p < 0.05, **p < 0.01, *** *p* < 0.001).

To explore IRF5 function in thyroid cancer cells, we employed lentiviral vectors to over-express this protein in SW1736, WRO, 8305C and C643. pLEX-IRF5-GFP variant 3 was generated by cloning the IRF5 cDNA fitted with SpeI-NotI adapters in the pLEX-MCS backbone (Open Biosystems, OHS4735). Transient transfection into TLA-HEK293 produced recombinant lentiviruses used to transduce thyroid cancer cells according to the Open Biosystems TLP4617 protocol. After 72 hours of puromycin selection (2.5 μg/mL), we performed an anti-IRF5 immunoblot to confirm transgene expression (Figure 3A) and 5 × 10^3^ resistant cells were placed in 96-well plates to perform ATP-Lite assays according to the manufacturer’s instructions (PerkinElmer). We found that IRF5 over-expression conferred a proliferative advantage to three of four thyroid cell lines (SW1736, WRO, and C643) suggesting a role for IRF5 in promoting thyroid cancer growth (Figure 3B). These results were further confirmed by a significant increase in the proliferation rate of SW1736 and C643 in doubling time experiments with a modest effect on WRO cells (Figure 3C).

**Figure 4:**
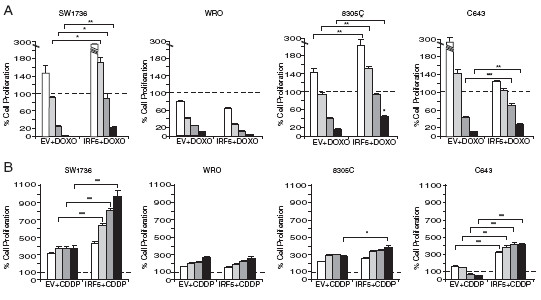
**IRF5 protects thyroid cancer cells from the effects of DNA-damaging agents. A.** The experiment described in Figure 3 was repeated comparing cell growth before and after exposure to 2 μM Doxorubicin (DOXO) or 5 μg/mL cis-diamminedichloroplatinum (CDDP) (both from Sigma). Columns represent average ± standard deviation of three independent experiments. *p-values* with 95% confidence intervals were calculated as described in the previous figure (*p < 0.05, **p < 0.01, *** *p* < 0.001).

We also wanted to assess if IRF5 protected neoplastic thyroid cells from the cytotoxic effects of two DNA-damaging compounds: doxorubicin (DOXO) and cis-diamminedichloroplatinum (CDDP). 5 × 10^3^ cell lines over-expressing IRF5 variant 3 were implanted in 96-well plates and left untreated or exposed to 2 μM DOXO or 5 μg/mL CDDP. After DOXO or CDDP treatment, ATP-Lite assays showed increased cell numbers in SW1736, 8305 C, and C643 transduced with IRF5 as compared to the EV control. IRF5 did not affect WRO cell responsiveness to either DOXO or CDDP (Figure 4A , B).

### IRF5 increases both clonogenic ability and B-raf expression in thyroid cancer cells

**Figure 5:**
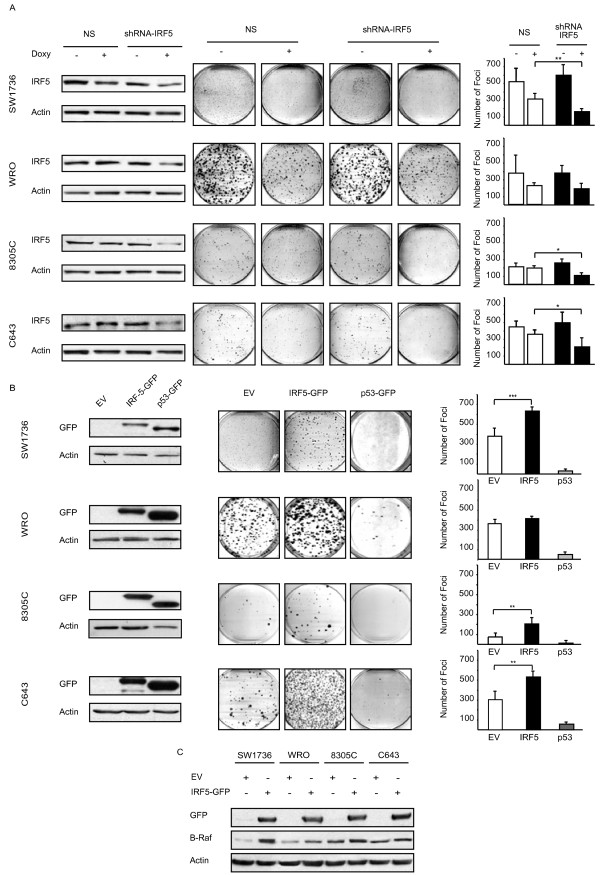
**IRF5 over-expression increases the foci-forming capacities of thyroid cancer cells. A.** Thyroid cells were infected with a doxycycline (Sigma) (Doxy)-inducible vector encoding either non-silencing or anti-IRF5 shRNA. Three days after doxycycline induction, lysates were blotted with an anti-IRF5 antibody to confirm reduction of IRF5 expression (left panels). After 1–2 weeks, infected cells were stained for their foci-forming ability (middle panels). Columns shown in the right panels represent average foci number in each plate ± standard deviation from three separate experiments (right panels). *p-values* were determined as reported in Figure 3. **B.** Thyroid cancer cells transduced with the specified constructs were analyzed by immunoblot for the expression of IRF5-GFP or p53-GFP (left panels). After 1–2 weeks cells were stained for their foci-forming ability (middle panels). Graphs depicted in the right panels represent average foci number in each plate ± standard deviation from three separate experiments. **C.** The same cells indicated in B, were analyzed for B-Raf (UPSTATE) expression by immunoblot.

To establish the tumor-promoting effect of endogenous IRF5 on thyroid cancer cells, we silenced its expression using the doxycicline inducible pTRIPZ vector (shRNA anti-IRF5, gene ID NM_001098627, Open Biosystems). A non-silencing shRNA (Cat. No. RHS4743) was employed as a control. Infected thyroid cancer cells were treated with doxycicline (1 μg/mL) for 72 hours, lysed and blotted for IRF5 to confirm reduced expression of the transcription factor. Clonogenic assays were then performed as described above with doxycicline added every 24 hours to maintain IRF5 silencing. Reduced IRF5 expression decreased thyroid cancer colony formation implying that, in thyroid cancer cells, IRF5 facilitates single cell growth (Figure 5A).

To confirm the increased clonogenic ability of cells over-expressing IRF5, we compared clones infected with either pLEX-IRF5-GFP or pLEX-p53-GFP (inserted in the unique XhoI site of pLEX-MCS). 1 × 10^3^ SW1736, WRO, C643 and 2 × 10^3^ 8305C were plated in a 100 mm dish and fresh medium was replaced every 72 hours for 10–14 days. At this time, colonies were stained with crystal violet (0.5% in 20% Ethanol/PBS) and counted. IRF5 over-expression induced a significant increase in the total number of foci generated by three of four cell lines, suggesting that this protein improves the colony-forming ability of individual thyroid cancer cells. As expected, p53 drastically reduced colony formation compared to the EV control (Figure 5B).

To investigate the molecular mechanism underlying the unexpected effects of IRF5, we evaluated B-Raf expression, as this protein is mutated in 30-40% of thyroid carcinomas [20]. Interestingly, ectopic IRF5 induced endogenous B-Raf levels in all thyroid cancer cells (Figure 5C).

## Discussion

In this study, we report - for the first time - that IRF5 is expressed in different thyroid carcinoma histotypes and in multiple thyroid cancer cell lines but is not detectable in normal human thyrocytes (Figure 1A). In primary normal and tumor thyroid cancer cells IFNα does not modulate IRF5 levels (Figure 1B) while in immortalized cell lines, IRF5 seems oddly responsive to IFNα, since exposure to this cytokine reduces IRF5 in two thyroid cancer cells and consistently increases its expression in one cell line (Figure 1C). We found both endogenous and ectopic IRF5 in the cytoplasm of thyroid cancer cells and IFNα was unable to relocate the protein in the nucleus (Figure 2A, B and Additional file 2). Not surprisingly, IRF5 did not induce the p21 promoter (Figure 2C, D) [17]. On the contrary, IRF5 stimulated thyroid cancer proliferation (Figure 3B, C), protected malignant thyroid cells from the cytotoxic effects of different antiblastic compounds (Figure 4), and significantly increased their colony-forming ability (Figure 5B). Indeed, silencing of endogenous IRF5 by lentiviral shRNA reduces the clonogenic potential of most thyroid cancer cells (Figure 5A). The induction of B-Raf expression detected in thyroid cells transduced with IRF5 could partially explain its tumor-promoting effects (Figure 5C).

These findings pose the pivotal question of what role is fulfilled by IRF5 in thyroid cancer. The initial observation that the IRF5 protein is not expressed in normal thyrocytes but is detected in neoplastic thyroid cells is somewhat surprising as IRF5 has been usually associated with tumor-suppressor rather than tumor-promoting activities [8,21,22]. Our finding that thyroid cancer cells localize IRF5 to the cell cytoplasm implies that the protein is transcriptionally inactive. Indeed, IRF5 fails to induce a previously characterized target such as p21 and does not arrest cell-cycle progression. On the contrary, IRF5 lentiviral over-expression significantly increases the proliferation rate of malignant thyroid cells. Taken together these findings suggest two alternative scenarios: a) IRF5 might be inactivated due to point mutations or increased tyrosine phosphorylation as previously reported [22]; b) IRF5 might be either inducing the expression of tumor-promoting genes or inhibiting the promoters for tumor-suppressors. Our supplemental data seem to exclude the first scenario as direct sequencing of IRF5 revealed no point mutations and we failed to observe any variations in IRF5 tyrosine phosphorylation levels (Additional file 3). The second hypothesis seems much more likely as suggested by: i) our findings showing increased B-Raf expression in thyroid cancer cells lentivirally transduced with IRF5; ii) the protective effect of IRF5 on thyroid cells exposed to different cytotoxic drugs; iii) the reduced colony-forming potential of malignant thyrocytes displaying reduced IRF5 levels.

In summary, the present study indicates that IRF5 favors the thyroid tumoral phenotype. However, the exact mechanisms underscoring this unexpected biological function remain partially unresolved.

## Abbreviations

IRF5, Interferon Regulatory Factor-5; IFNα, Interferon-alpha; GFP, Green Fluorescence Protein; Luc, Luciferase; EV, Empty Vector; MCS, Multiple Cloning Site; DOXO, Doxorubicine; CDDP, Cis-diamminedichloroplatinum; shRNA, Short-Hairpin RNA; Doxy, Doxycicline.

## Competing interests

The authors declare that they have no competing interests.

## Authors’ contributions

MM, MF, AF and AA carried out the research. FF participated in coordination and data analysis. LM and PV designed the study and drafted the manuscript. All authors read and approved the final manuscript.

## Supplementary Material

Additional file 1 **IRF5 shows cytoplasmic localization in thyroid cancer cells after IFN**a **treatment.** The specified cell lines were treated with 1000 U/mL IFNa for 24 hours. Cells were then labeled for IRF5 using the indicated secondary antibody. Hoechst and phalloidin were employed to stain nuclear and cytoplasmic compartments. Click here for file

Additional file 2 **IRF5 v3 shows cytoplasmic localization in thyroid cancer cells.** The specified cell lines were lentivirally infected with IRF5-GFP v3 and the proteins intracellular localization was analyzed by immunofluorescence. Click here for file

Additional file 3 **IRF5 tyrosine phosphorylation is not modulated by DNA-damaging agents in thyroid cancer cells.** Whole lysates of the specified cell lines treated with 2 μM Doxorubicin (DOXO) or 5 μg/mL cisdiamminedichloroplatinum (CDDP) for the indicated times were subjected to an anti-phosphotyrosine (pY) immunoprecipitaion and subsequently blotted for IRF-5. G immunoglobulines (IgG) were used as a loading control. Click here for file
