# Supplementary material for: IRF5 promotes the proliferation of human thyroid cancer cells
Source: Mol Cancer. 2012 Apr 16;11:21. doi: 10.1186/1476-4598-11-21 (PMC3444366; doi:10.1186/1476-4598-11-21)
Supplement: Additional file 1 — IRF5 shows cytoplasmic localization in thyroid cancer cells after IFNa treatment. The specified cell lines were treated with 1000 U/mL IFNa for 24 hours. Cells were then labeled for IRF5 using the indicated secondary antibody. Hoechst and phalloidin were employed to stain nuclear and cytoplasmic compartments. [file 1476-4598-11-21-S1.pdf]

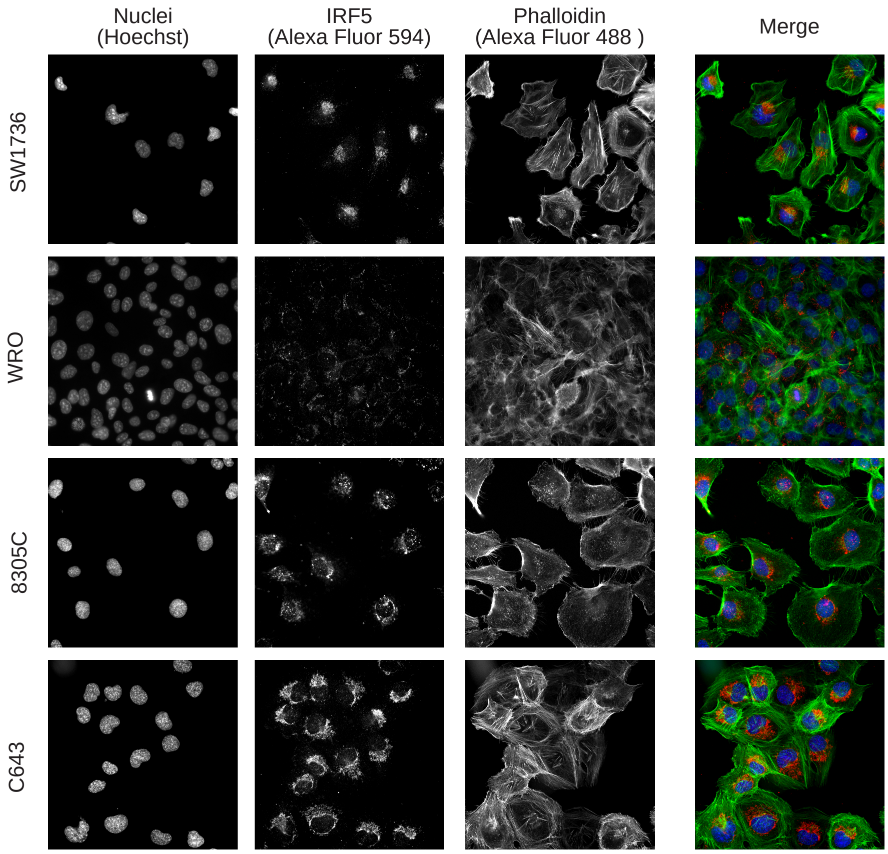

Additional file 1. **IRF5 shows cytoplasmic localization in thyroid cancer cells after IFNα treatment.** The specified cell lines were treated with 1000 U/mL IFNα for 24 hours. Cells were then labeled for IRF5 using the indicated secondary antibody. Hoechst and phalloidin were employed to stain nuclear and cytoplasmic compartments.
