# Supplementary material for: IRF5 promotes the proliferation of human thyroid cancer cells
Source: Mol Cancer. 2012 Apr 16;11:21. doi: 10.1186/1476-4598-11-21 (PMC3444366; doi:10.1186/1476-4598-11-21)
Supplement: Additional file 2 — IRF5 v3 shows cytoplasmic localization in thyroid cancer cells. The specified cell lines were lentivirally infected with IRF5-GFP v3 and the proteins intracellular localization was analyzed by immunofluorescence. [file 1476-4598-11-21-S2.pdf]

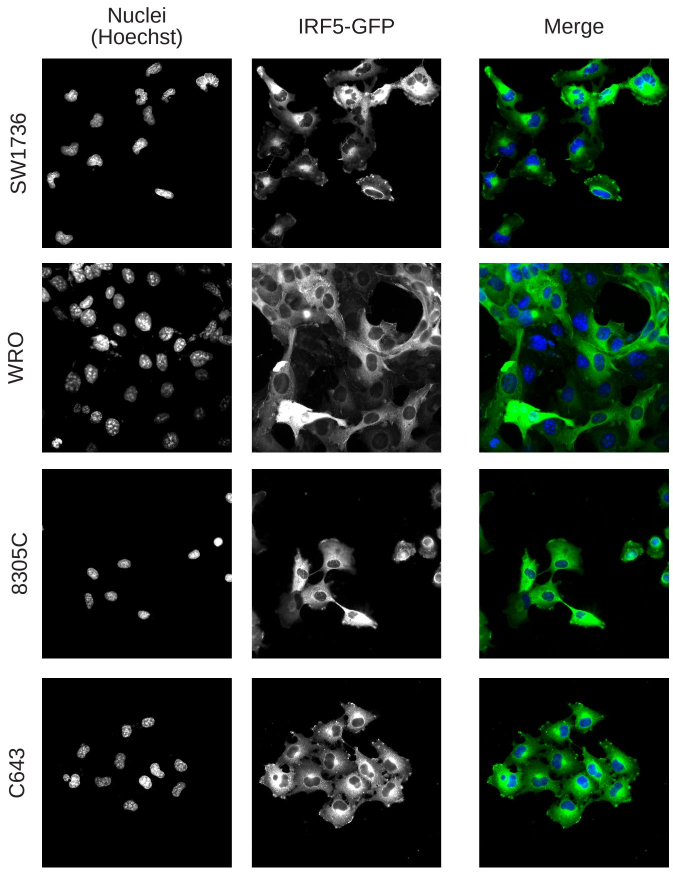

Additional file 2. **IRF5 v3 shows cytoplasmic localization in thyroid cancer cells.** The specified cell lines were lentivirally infected with IRF5-GFP v3 and its intracellular localization was analyzed by immunofluorescence.
