# Supplementary material for: IRF5 promotes the proliferation of human thyroid cancer cells
Source: Mol Cancer. 2012 Apr 16;11:21. doi: 10.1186/1476-4598-11-21 (PMC3444366; doi:10.1186/1476-4598-11-21)
Supplement: Additional file 3 — IRF5 tyrosine phosphorylation is not modulated by DNA-damaging agents in thyroid cancer cells. Whole lysates of the specified cell lines treated with 2 μM Doxorubicin (DOXO) or 5 μg/mL cisdiamminedichloroplatinum (CDDP) for the indicated times were subjected to an anti-phosphotyrosine (pY) immunoprecipitaion and subsequently blotted for IRF-5. G immunoglobulines (IgG) were used as a loading control. [file 1476-4598-11-21-S3.pdf]

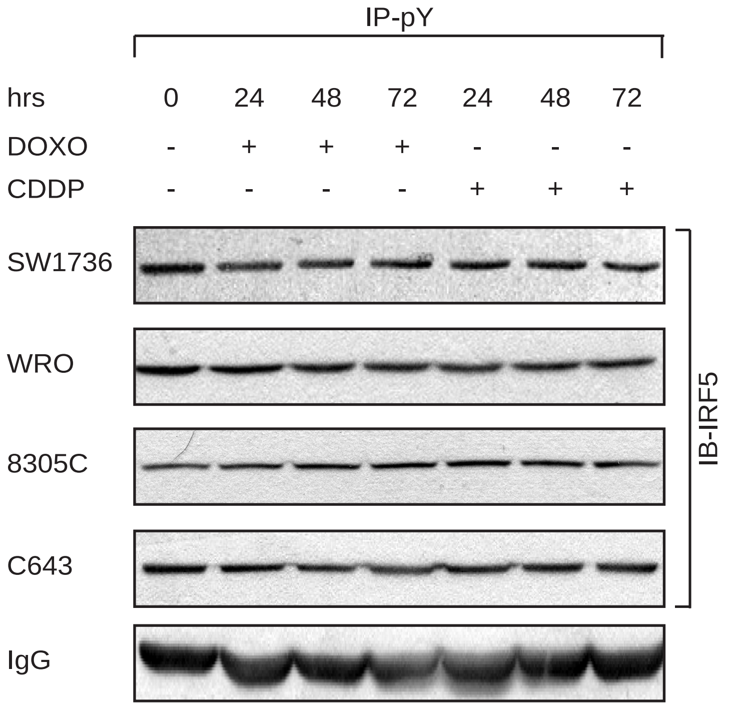

Additional file 3. IRF5 tyrosine phosphorylation is not modulate from DNA-damage agents treatment in thyroid cancer cells.

The specified cell lines were treated with 2µM Doxorubicin (Dox) or 5µg/mL cis-diamminedichloroplatinum (CDDP) for indicate time. An immunoprecipitaion anti-pY was perform, followed by immunoblot anti-IRF-5. The immunoglobuline (IgG) was used as loading control.
